# Treatment of Root Canals

**Published:** 1901-08-15

**Authors:** Wm. E. Harper

**Affiliations:** Chicago, Ills.


### TREATMENT OF ROOT CANALS.

BY WM. E. HARPER, D.D.S., CHICAGO, ILLS.

Read before the Southwestern Michigan Dental Society.

*Mr. President and Gentlemen:*—In the presentation of this subject this morning I thought it would be of greater interest, or at least bring it to your minds more clearly, if I should describe an operation from beginning to end, giving the details in all parts of the operation which I consider essential to success. I shall not preach anything I do not practice.

Taking a case in which devitalization is considered necessary, the first essential point is the opening of cavity, which is best accomplished by cleaving down enamel, dispensing with the use of the engine. Do not simply open it up so as to allow a small amount of arsenic to be placed therein but make large enough opening to see what you are doing, without trying to remove pulp. Extend to buccal, lingual and occlusal and remove all decay from surrounding walls of cavity; this is imperative to enable you to seal in the arsenic. After doing this then apply rubber dam and with large spoon excavator, as large as cavity will permit, loosen up edges of the softened carious dentin over the pulp; use large spoon excavator so it will not slip into the tissues of the pulp. The cutting edge of instrument should always be larger than the diameter of the pulp chamber. Scrape out decay from pulp with long diameter of pulp chamber. By resting one portion of blade of excavator on the incisal wall of the pulp chamber will prevent it from entering and injuring the pulp, this as you know is often the cause of great pain, and is simply due to lack of attention to this little detail. With pulp exposed in this manner make application of arsenic and seal. I seal preferably with gutta-percha, because cements are not water-tight. After the application is made you may dismiss patient for a convenient length of time, whether for twenty-four hours or seven days is a matter of convenience or preference. I make the second appointment at the most convenient time, any time after twenty-four hours, must seal in arsenic permanently, however.

At second sitting after applying rubber dam before removal of filling, swab the entire field of operation, both rubber dam and all teeth included in the dam, with deodorized oil of cloves or Black's 1-2-3 remedy so as to sterilize the entire field. Then remove filling and at this time again wash out cavity with antiseptic; I use oil of cloves altogether.

At this time the finished cavity should be practically prepared, all the extensions necessary to meet requirements of extension for prevention should be made; in other words, the cavity should be prepared before opening into the pulp chamber, cleaning out all decay, and even making retention form. The cavity should again be washed with oil of cloves and made as sterile as possible.

The tooth now being ready the operator's fingers should be sterilized, as well as all instruments which enter the pulp chamber. If the pulp is completely devitalized, open the chamber with small, round bur getting absolute and direct access into root canals. Do this in molars and bicuspids by cutting away the entire occlusal wall of pulp chamber, make the walls of pulp chamber continuous with walls of cavity if it is a distal cavity, in bicuspids and molars this cutting away must be carried to mesial marginal ridge. We can not treat these cases with any degree of certainty unless the entire middle third of buccal or lingual wall, as far as the mesial marginal ridge, is cut away, because the mesial buccal canal projects more toward the mesial buccal angle of tooth in upper molars. This will insure a more perfect operation and facilitates instrumentation. In lower molars cut to mesial marginal ridge, but extend wall to the buccal on account of inclination of buccal surface to the lingual. We often find in the mesial roots of lower first molars two canals, there is a slit-like connection between the two. Many fail to extend the opening sufficiently upon the occlusal, and in this way fail to get into the buccal portion of the canal, and therefore leave it unfilled; they cut into center of sulcus and forget all about the inclination of tooth; the buccal canal in mesial root is directly under mesial buccal cusp, but we can not approach it from sulcus but must extend to mesial buccal cusp to get access. In making complete preparation of cavity first, we avoid the danger of forcing pieces of decayed dentin into the canals. Prepare the cavity first, and then open into canals.

The removal of pulp should first be attempted with a barbed broach. Before using a broach test it by placing a finger on the end and bend it back and forth, oftentimes in cutting the barbs on broaches one is cut too deep, in this way weakening it, if there is such a place in a broach it will break under this manipulation. If it is perfectly regular it will bend in an even curve and is all right to use. Having tested the broach dip it in oil of cloves, and then carry it against one of the walls of the root canal into the canal as far as possible toward the apex; having reached this point, then withdraw it a very little distance, careful observation of this point will save the breaking of a great many broaches. The point of the broach which is carried into the foramina is broken off many times by rotating it when fast in the dentin, by withdrawing it a small distance this accident is averted. Now rotate broach just once and then withdraw, place in again after the above-mentioned manner and rotate once and withdraw again, in many cases the pulp will be removed entire. Failing to do so after this manner take a smooth piano wire broach, file down with a coarse file, the surface is then rough enough to hold a few fibers of cotton. This is carried into the root canal as far as apex, then rotated a few times and withdrawn. If the pulp has been disintegrated by a barbed broach this is a very effective method for removal of the schreds. After the pulp has been entirely removed wipe dry as possible with cotton. I absolutely object in my office to the use of warm air or the root drier, am perfectly satisfied to wipe canals dry and then proceed to fill. If the canals need to be enlarged for successful filling do so at this time, using for this purpose a barbed broach. Many operators attempt to enlarge root canals by rotating a barbed broach. It should be used as a mechanic uses a file, it cuts only as you pull and does not cut in passing into the canal. Enlargement is accomplished by increasing the size of the broaches. There is no quicker method of enlarging root canals than this. After cleaning away the debris, wipe the canal with eucalyptol, it has great affinity for the organic substances of the tooth and displaces the moisture, and has a solvent action on the gutta-percha filling material causing it to adhere to the walls of the canal. Don't saturate but just moisten the canals with eucalyptol. Have several root canal pluggers of different sizes made of metal which is very flexible. Select one which just goes into the canal, heat the end and touch to it a small end of gutta-percha point, four or five millimeters long, pass it into the canal and force into place, it will be held by the walls of the canal and pushed ahead of the plugger. In placing a large piece, if there is any projection in the canal, it will buckle and fail to fill the canal. Packing small pieces at a time and carrying each one carefully ahead on a small plugger, and packing bit by bit we may be assured that it is packed solidly and is as near perfectly done as possible. After packing the apical half take a larger root canal plugger and pack the remainder to orifice of canal. I never fill the pulp chamber with gutta-percha as I want the floor of pulp chamber for retention form. If it be an amalgam filling I complete it at the same sitting.

The question will naturally arise, What are we going to do with those canals which are too small to receive root canal pluggers? If we can get the smallest Donaldson broach into a canal, we can enlarge in the manner above referred to. If the canals are too small to receive a barbed broach attempt to pass a smooth broach saturated with an antiseptic, pass it into the canal as far as possible and you will often find it will go as far as the apex. With the smooth broach try and pump into the canal the antiseptics in some degree if possible. Remove any small portion of organic matter which is possible under the circumstances, which does not generally amount to much. Work into the canal eucalyptol, then a small amount of chloro-percha is placed over orifice of canal and by using the small broach as much as possible is pumped in. Take a copper point, not because of antiseptic properties, but because it can be made into such delicate cones or points and fairly stiff, and still not so much so as to prevent it filling the tortuous canals. If the patient is conscious of the passage of the point withdraw and cut off the end and reinsert. We do not want any metallic cone to project beyond the apex.

One thing to avoid in the opening of pulp chambers is the marring of the floor of the pulp chamber with the bur, the floor of the chamber slants off in the various canals in a conical shape thus guiding you into canal.

In the lower first molars, particularly, the bifurcation of roots is much closer to the gingival portion of tooth than in any other tooth in the mouth. Many in searching for these canals drill through the bifurcation.

In reference to the drying out process never use heat, because the enamel is easily checked by its use. Because of the different amounts of organic material in the dentin and enamel, the shrinkage is different and enamel is checked. I am convinced that an immense amount of damage is done by this manipulation. Dr. Black, in his work upon teeth, found that teeth checked if allowed to stand in air ten minutes after extracting from mouth. Now, if enamel can be checked by the dissipation of so small an amount of moisture as that, is it not reasonable to suppose that drying out with heat when you have the crown of a tooth excluded from moisture by the use of rubber dam, and in blowing in a blast of air from the root dryer you take up more moisture from tooth than is best for it? Proceeding as we have suggested, if moisture is present it is made sterile, and nothing is gained by drying it out.

### DISCUSSION.

DR. RUNYAN: I am sure we are all very thankful to Dr. Harper for the suggestions he has given us. Many of us will be benefited and see wherein we have failed in getting access to the root canals. The manner of operating, particularly the suggestion for instrumentation in uncovering the pulp is a remarkably good one. We have all had more or less trouble along that line and also in the matter of securing asepsis. The oil of cloves is a very simple antiseptic and one of the best for the purpose. The suggestion in reference to leaving the nerve in root canal until the cavity is prepared is also a good one.

I would like to ask Dr. Harper if he prepares the copper points, if not, where they can be bought? I should think they would be a great aid in the filling of the small canals. The paper is full of good suggestions, and these are the ones which have enlisted my interest.

Dr. Watling, of Ypsilanti, Mich.: I feel as though I ought to take issue with Dr. Harper on the question of warm air. Have had considerable experience with the use of it. There is no doubt that absolute dryness is one of the best antiseptics. If the essayist's statement in reference to the drying of teeth holds, the rubber dam has been one of the meanest and most objectionable inventions brought into the profession, for it excludes moisture from the teeth and exposes them to the dry air for several hours at a time on some occasions.

I will say, however, that judgment should be exercised with the use of warm air. If used correctly it will cause slight absorption or expression of moisture. I never think of blowing *hot* air but just *warm*, and I have a specially constructed hot air syringe which I have used for years. I have seen operators, and especially students, use the same syringe for hot air as they use for a chip blower; they heat it over the alcohol lamp and blow into tooth cavity. If you take that same air and blow onto a window pane you will find it always contains moisture, for it will condense upon the glass. A properly made warm air syringe will not do so, the air in it contains no moisture.

Dr. Taft, of Cincinnati, Ohio: I hesitate to say much upon this subject. While the chief trend of Dr. Harper's remarks are correct, there are some things of course to which exception might be taken; Dr.

Watling has mentioned one. That the use of warm air could by any means tend to check the enamel is something very strange to me, however there are a great many things which are true that are not always clear to me. As Dr. Watling has mentioned, the exclusion of moisture from teeth by the rubber dam would be very disastrous to a tooth if this theory is correct. We find a great many teeth in the mouth which are checked that have never been subjected to such treatment.

The use of warm air is an historical matter. The idea first originated with Dr. Townsend, who, in conversation with Dr. Watt, remarked that if there was some way of throwing a jet of warm air into a cavity it would be an excellent thing. From that conversation Dr. Geo. Watt constructed the first warm air blow pipe.

I fully concur with the remarks of Dr. Harper in reference to it not being necessary to have absolute dryness; that is, to have all the moisture removed from the canals of dentin, but simply blowing in hot air would not do this. Alcohol will take out water if necessary, but I can see no objection to the use of warm air when properly applied.

The average operator will not take the time for the work as outlined for this operation by Dr. Harper; however, the time used in this way should be well repaid in a successful operation.

Dr. Warboys, of Albion: There is one thing about which I wish to speak particularly to the dentists of the smaller towns. We are liable to do this class of work for too small a fee. Therefore we do not spend the proper time or effort on it. We can not get the fee for the length of time which it takes to properly do this; it does not show up to the patient like a large gold filling.

Dr. Fowler: I would like to ask Dr. Harper what he uses to devitalize the pulp?

Dr. Harper: I use both arsenic and pressure anesthesia. If I use arsenic I do not hesitate to leave it in for several days. I do not make the copper points, the S. S. White Company makes them, also the root canal pluggers of which I spoke.

Referring to the drying question, I do not mean that regular cracks form in the enamel, neither do I mean that you injure the enamel, or predispose it to decay, but after such treatment that tooth will not stand more than three-fourths as much strain as it would before. For proof of this subject will refer you to Dr. Black's experiments on physical aspects of teeth. It has also been said that this operation takes more time. I do not think my time wasted in this operation, it takes me about one-half hour to do it and I can point you to operators who spend an hour doing it.

You can readily displace water from a root canal by alcohol and eucalyptol, the plasmic elements in the tubuli I would just as soon have remain if sterile.

So, do not think I believe this operation predisposes a tooth to decay but it simply weakens it, and since just as successful an operation can be made without the use of hot air, why use it?

Dr. Higgins: Do you remove the pulp and fill the root canals at one sitting, especially where you use the arsenic?

Dr. Harper: Where I use arsenic I usually fill the root canal immediately, and if an amalgam filling I fill the crown; have done this for eight years with success. A pulp devitalized with arsenic is in an aseptic condition. If I devitalize a pulp and carry out the operation under the conditions before outlined I am ready to fill it immediately.

DR. FOWLER: If there is soreness of the tooth do you fill at same sitting?

DR. HARPER: I do not usually have a condition of soreness; of course, inflammation of the apical space would forbid immediate filling. In slight soreness the only reason I would defer would be in consideration for the patient, for if you fill such a tooth it will subside in a short time; oil of cassia makes a tooth very sore at times.

DR. DOUGLASS: In case of hemorrhage what do you do?

DR. HARPER: In this case the most satisfactory method which I have tried is to take a broach and dip into carbolic acid and allow it to remain there five or six minutes. If I can stop the hemorrhage I then fill as above mentioned. I use oil of cloves for everything, even sharpen my instruments, using oil of cloves as a lubricant on the stone.

